# Novel Approaches to Improve the Efficacy of Immuno-Radiotherapy

**DOI:** 10.3389/fonc.2019.00156

**Published:** 2019-03-19

**Authors:** Maxim Shevtsov, Hiro Sato, Gabriele Multhoff, Atsushi Shibata

**Affiliations:** ^1^Center for Translational Cancer Research, Technical University of Munich, Klinikum rechts der Isar, Munich, Germany; ^2^Institute of Cytology, Russian Academy of Sciences (RAS), St. Petersburg, Russia; ^3^First Pavlov State Medical University of St. Petersburg, St. Petersburg, Russia; ^4^Almazov National Medical Research Centre, Polenov Russian Scientific Research Institute of Neurosurgery, St. Petersburg, Russia; ^5^Department of Radiation Oncology, Graduate School of Medicine, Gunma University, Maebashi, Japan; ^6^Education and Research Support Center, Graduate School of Medicine, Gunma University, Maebashi, Japan

**Keywords:** radiotherapy, immunosuppression, immune checkpoint inhibitors, PD-L1, PD-1

## Abstract

Radiotherapy (RT) has been applied for decades as a treatment modality in the management of various types of cancer. Ionizing radiation induces tumor cell death, which in turn can either elicit protective anti-tumor immune responses or immunosuppression in the tumor micromilieu that contributes to local tumor recurrence. Immunosuppression is frequently accompanied by the attraction of immunosuppressive cells such as myeloid-derived suppressor cells (MDSCs), M2 tumor-associated macrophages (TAMs), T regulatory cells (Tregs), N2 neutrophils, and by the release of immunosuppressive cytokines (TGF-β, IL-10) and chemokines. Immune checkpoint pathways, particularly of the PD-1/PD-L1 axis, have been determined as key regulators of cancer immune escape. While IFN-dependent upregulation of PD-L1 has been extensively investigated, up-to-date studies indicated the importance of DNA damage signaling in the regulation of PD-L1 expression following RT. DNA damage dependent PD-L1 expression is upregulated by ATM/ATR/Chk1 kinase activities and cGAS/STING-dependent pathway, proving the role of DNA damage signaling in PD-L1 induced expression. Checkpoint blockade immunotherapies (i.e., application of anti-PD-1 and anti-PD-L1 antibodies) combined with RT were shown to significantly improve the objective response rates in therapy of various primary and metastatic malignancies. Further improvements in the therapeutic potential of RT are based on combinations of RT with other immunotherapeutic approaches including vaccines, cytokines and cytokine inducers, and an adoptive immune cell transfer (DCs, NK cells, T cells). In the current review we provide immunological rationale for a combination of RT with various immunotherapies as well as analysis of the emerging preclinical evidences for these therapies.

## Immunosuppressive Effects of Radiotherapy (RT)

**Figure 1:**
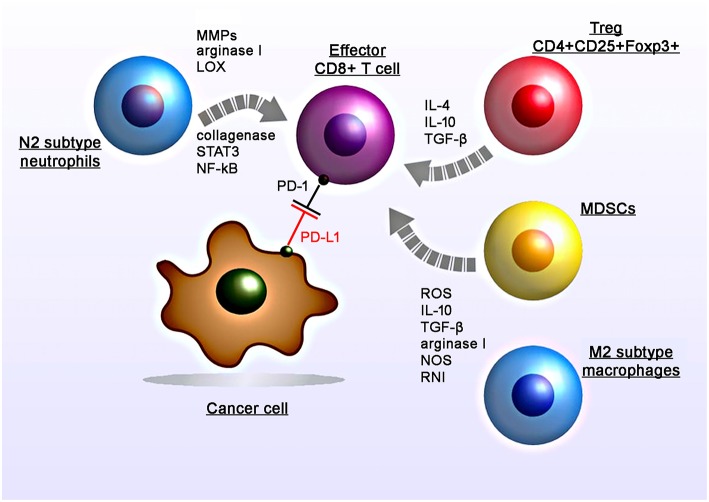
Radiation-induced immunosuppressive effects in the tumor micromilieu. RT induces recruitment, proliferation and polarization of immunosuppressive cell subtypes including myeloid-derived suppressor cells (MDSCs), M2 tumor-associated macrophages, N2 neutrophils, and regulatory T cells (CD4+CD25+Foxp3+). RT induces increased levels of suppressive factors including nitric oxide synthase (NOS) and reactive nitrogen intermediates (RNI), reactive oxygen species (ROS), cytokines (IL-4, IL-10, TGF-β), matrix metalloproteinases (MMPs), arginase I, collagenase, lipoxygenase (LOX) which in turn leads to the suppression of the T cell activity.

Apart from surgery and chemotherapy, RT is one of the major pillars to treat solid tumors. However, the radiation-induced immunosuppression could hamper the therapeutic benefits of abscopal effects (Figure 1). RT can contribute to the anti-immunogenic micromilieu by recruiting TAMs and MDSCs (1–3). Previously Ahn et al. demonstrated that irradiated tumors recruit large numbers of bone marrow-derived CD11b+ myeloid suppressor cells that express matrix mettaloproteinase-9 (MMP-9) which can promote tumor growth and metastatic spread (4). Proangiogenic effects are induced by the expression of various chemoattractants and angiogenic molecules including Bv8, S100A8, TGF-β, and VEGF (5–8). Furthermore, production of arginase I by MDSCs decreases the expression of the zeta chain of the CD3 complex (CD3ζ) and thereby impairs T cell activity (9). Intriguingly, as recently shown by Noh et al. MDSCs could also induce the expression of PD-L1 on tumor cells (10).

Subsequent studies demonstrated that the application of anti-CD11b antibodies in human squamous cell carcinomas in mice resulted in a reduced tumor-infiltration of MDSCs (expressing S100A8 and MMP-9) and thereby increases the efficacy of radiation (1). Another study by Crittenden et al. showed that following radiation therapy influx of M2-differentiated macrophages into the tumor stroma induced immune suppression mediated by a transcriptional regulation of NFκB p50 (3). Radiation-induced apoptotic cancer cells activate M2 macrophages that in turn secrete various anti-inflammatory cytokines including TGF-β and IL-10 (11, 12).

Among other immune cells also Tregs have been found to be enriched in the tumor after irradiation (13). Tregs (CD4+CD25+Foxp3+) play a major role in regulating anti-tumor immune responses via direct cell-to-cell contacts and the release of various cytokines such as TGF-β, IL-4, IL-10 (14, 15).

Irradiated tumors could also recruit large numbers of CD11b+Gr1+ neutrophils (16). The presence of TGF-β in the tumor micromilieu induces the promotion of pro-tumoral N2 neutrophils and the activation of PI3K-Akt, RHOA, MAPK, and SMAD pathways (17, 18). Indeed preclinical studies demonstrated that anti-Ly6G antibodies that deplete neutrophils can improve the efficacy of RT (19). In contrast, the application of anti-Gr1+ antibodies did not affect radiation-induced immunity (1).

Additionally irradiation induces an upregulation of the PD-L1 expression on tumor cells which in turn blocks the function of activated T and NK cells against tumors (20). Studies analyzing PD-L1 expression on tumor cells following irradiation also demonstrated that this upregulation could be mediated by IFNγ, which is produced by T cells (21). Particularly conventional (5 × 2 Gy) and hypo-fractionated but not single high dose irradiation increases the surface expression of PD-L1 on melanoma B16-F10 and glioblastoma GL261 cells, *in vitro* (22). Furthermore, standard RT combined with chemotherapy increased the expression of PD-1 on CD4+ T cells in the peripheral blood in oropharyngeal cancer patients (23).

Among other immunosuppressive chemokines and cytokines hypoxia-inducible factor-1α (HIF-1 α), adenosine, lactate, potassium, vascular endothelial growth factor (VEGF), and acidosis have been found to block anti-tumor immune responses (24–26). Presumably, all mechanisms of radiation-induced immunosuppression [i.e., infiltration by MSCDs, Tregs, M2 macrophages, expression of inhibitory molecules (PD-L1)] represent cellular responses that constrain local tissue damage. The interference of these mechanisms particularly that of the immune checkpoint inhibitor axis could provide a promising strategy to further induce cancer cell damage via an activation of T and NK cell mediated anti-tumor responses.

## Immunotherapy in Combination With Cancer Therapy Causing DNA Damage Response

### Immune Checkpoint Inhibition

Evidence accumulated over the past decade that multiple factors are involved in the establishment of an immunosuppressive micromilieu of tumors (27, 28). For example defects in T cell receptor signaling, tumor-induced impairment of antigen presentation, activation of negative co-stimulatory signals, such as CTLA-4/CD80 (or CTLA-4/CD86) and PD-1/PD-L1, elaboration of immunosuppressive factors (IL-10, TGF-β, galectin-1, gangliosides, and PGE2), inactivation of pro-apoptotic pathways (FasL, TRAIL, IDO, and RCAS1), inhibition of natural killer (NK) cell mediated cytotoxicity, and inhibition of differentiation and maturation of dendritic cell (DC) have been found to establish an immunosuppressive environment that promotes tumor growth (29). The interference of the PD-1/PD-L1 and CLTA-4/CD80 (or CTLA-4/CD86) pathways has shown promising results in therapy of cancer of different entities (30). For example, ipilimumab which is an anti-CTLA-4 antibody, was approved by the US Food and Drug Administration (FDA) for the treatment of melanoma, advanced renal cell carcinoma, and metastatic colorectal carcinoma with high microsatellite instability (MSI) or mismatch repair (MMR) deficiencies (Table 1). Nivolumab, targeting PD-1 on T and NK cells was also approved by the FDA for the treatment of many types of cancers, including advanced or metastatic melanoma and metastatic, refractory non-small cell lung cancer (NSCLC) (Table 1) (31–35). These immune checkpoint inhibitor therapies restore anti-tumor immune responses by disrupting the interactions between receptors (PD-1 or CTLA-4) on T and NK cells and their corresponding ligands, PD-L1 on tumor cells or CD80/86 on antigen presenting cells, respectively. These immune checkpoint inhibition therapies provide effective anti-tumor effects by augmenting the body's own immune system against cancer (36, 37). However, although the predicted mechanism of the restoration of immune activity is attractive, patient responses are highly variable. For example, anti-PD-1/PD-L1 therapies result in impressive response rates in ~5% of the patients, whereas ~40% of the patients show cancer progression (31–35). Therefore, researchers are highly interested to improve therapeutic efficacy by identifying reliable biomarkers that could predict responses to an anti-PD-1/PD-L1 therapy (38). Although PD-L1 expression on tumor cells appears to be ideal for determining the efficacy of an anti-PD-1/PD-L1 therapy, its predictive quality is under debate, presumably due to various other factors that contribute to the immunosuppressive environment on an individual tumor. Thus, an improved understanding of the molecular mechanisms underlying the regulation of the PD-L1 expression in cancer cells is critical for the identification of valuable biomarkers for a personalization of an anti-PD-1/PD-L1 therapy. Another aspect refers to the identification of the best combination therapy (i.e., RT, chemotherapy, and molecular targeted drugs), which will be supportive for an anti-PD-1/PD-L1 therapy. However, despite promising results from *in vivo*- or clinical trials-based studies, understanding of the molecular mechanisms underlying the PD-L1 expression in cancer cells has not been completely elucidated.

**Table 1:** List of clinical trials in the FDA-approved Nivolumab and Ipilimumab.

**NIVOLUMAB (OPDIVO)**			
**Primary diagnosis**	**Details**	**Base clinical trials**	**Phase**
Melanoma	Unresectable or metastatic melanoma, Previously treated with ipilimumab and, if BRAF V600 mutation positive, a BRAF inhibitor	NCT01721746 (CHECKMATE-037)	3
	Previously untreated unresectable or metastatic melanoma	NCT01721772 (CHECKMATE-066)	3
		NCT01844505 (CHECKMATE-067)	3
	Adjuvant setting for lymph node involvement or metastatic after complete resection	NCT02388906 (CHECKMATE-238)	3
Non-small cell cancer	Squamous NSCLC metastatic with progression, on or after platinum-based chemotherapy, or FDA-approved therapy for EGFR or ALK genomic tumor aberrations for patients with these aberrations	NCT01642004 (CHECKMATE-017)	3
	Non-squamous NSCLC metastatic with progression, on or after platinum-based chemotherapy, or FDA-approved therapy for EGFR or ALK genomic tumor aberrations for patients with these aberrations	NCT01673867 (CHECKMATE-057)	3
Small cell lung cancer	Metastatic with progression, after platinum-based chemotherapy and at least one other line of therapy.	NCT01928394 (CHECKMATE-032)	1/2
Renal cell cancer	Advanced, after prior anti-angiogenic therapy	NCT01668784 (CHECKMATE-025)	3
	Previously untreated advanced intermediate or poor risk	NCT02231749 (CHECKMATE-214)	3
Classical Hodgkin lymphoma	Relapsed or progressed after 1. autologous hematopoietic stem cell transplantation (HSCT) and brentuximab vedotin, or 2. three or more lines of systemic therapy that includes autologous HSCT	NCT02181738 (CHECKMATE-205) NCT01592370 (CHECKMATE-039)	2 1/2
Head and Neck Squamous Cell Carcinoma	Recurrent or metastatic with progression, on or after a platinum-based therapy	NCT02105636 (CHECKMATE-141)	3
Urotherial carcinoma	Locally advanced or metastatic after 1. disease progression during or following platinum-containing chemotherapy, or 2. disease progression within 12 months of neoadjuvant or adjuvant treatment with platinum-containing chemotherapy.	NCT0238799 (CHECKMATE-275)	2
Colorectal cancer	Microsatellite instability-high (MSI-H) or mismatch repair deficient (dMMR) metastatic with progression, after fluoropyrimidine, oxaliplatin, and irinotecan	NCT02060188 (CHECKMATE-142)	2
Hepatocellular carcinoma	Previously treated with sorafenib	NCT01658878 (CHECKMATE-040)	1/2
**IPILIMUMAB (YERVOY)**			
Melanoma	Unresectable or metastatic melanoma	NCT00094653	3
	Adjuvant setting for cutaneous melanoma with pathologic involvement of regional lymph nodes of more than 1 mm who have undergone complete resection with total lymphadenectomy	NCT00636168	3
Renal cell cancer	Previously untreated advanced intermediate or poor risk	NCT02231749 (CHECKMATE-214)	3
Colorectal cancer	MSI-H or dMMR metastatic with progression, after fluoropyrimidine, oxaliplatin, and irinotecan	NCT02060188 (CHECKMATE-142)	2

Multiple studies have provided evidence that the PD-L1 expression is upregulated in cancer cells following RT or chemotherapy (21, 39, 40). Similar to RT, platinum-based drugs and alkylating agents induce DNA damage in cancer cells. Therefore, it is important to understand the mechanistic linkage between DNA damage signaling and PD-L1 expression.

### PD-L1 Expression Induced by Mutational Loads

PD-L1 expression in tumors is one important factor in the establishment of an immunosuppressive environment. Clinical data indicate that a high PD-L1 expression on tumors is associated with poor prognosis (41–43). The PD-L1 expression in tumors, as well as immune cells surrounding the tumor, can affect the efficacy of anti-PD-1/PD-L1 therapy (44, 45). In the regulation of PD-L1 expression in tumors, IFN is considered as a critical molecule to induce PD-L1 upregulation at the transcriptional level. Recent studies have revealed that type I IFN (α and β) and type II IFN (γ) cause PD-L1 upregulation in cancer and immune cells (46). Although all IFN subtypes are able to upregulate PD-L1, IFNγ exhibits a stronger and more prolonged effect than the type I IFNs (47). IFNγ binds to the IFNγ receptor and continuously stimulates downstream signaling of JAK1/2, STAT1/2/3, and IRF1 to induce the PD-L1 expression (47, 48).

**Figure 2:**
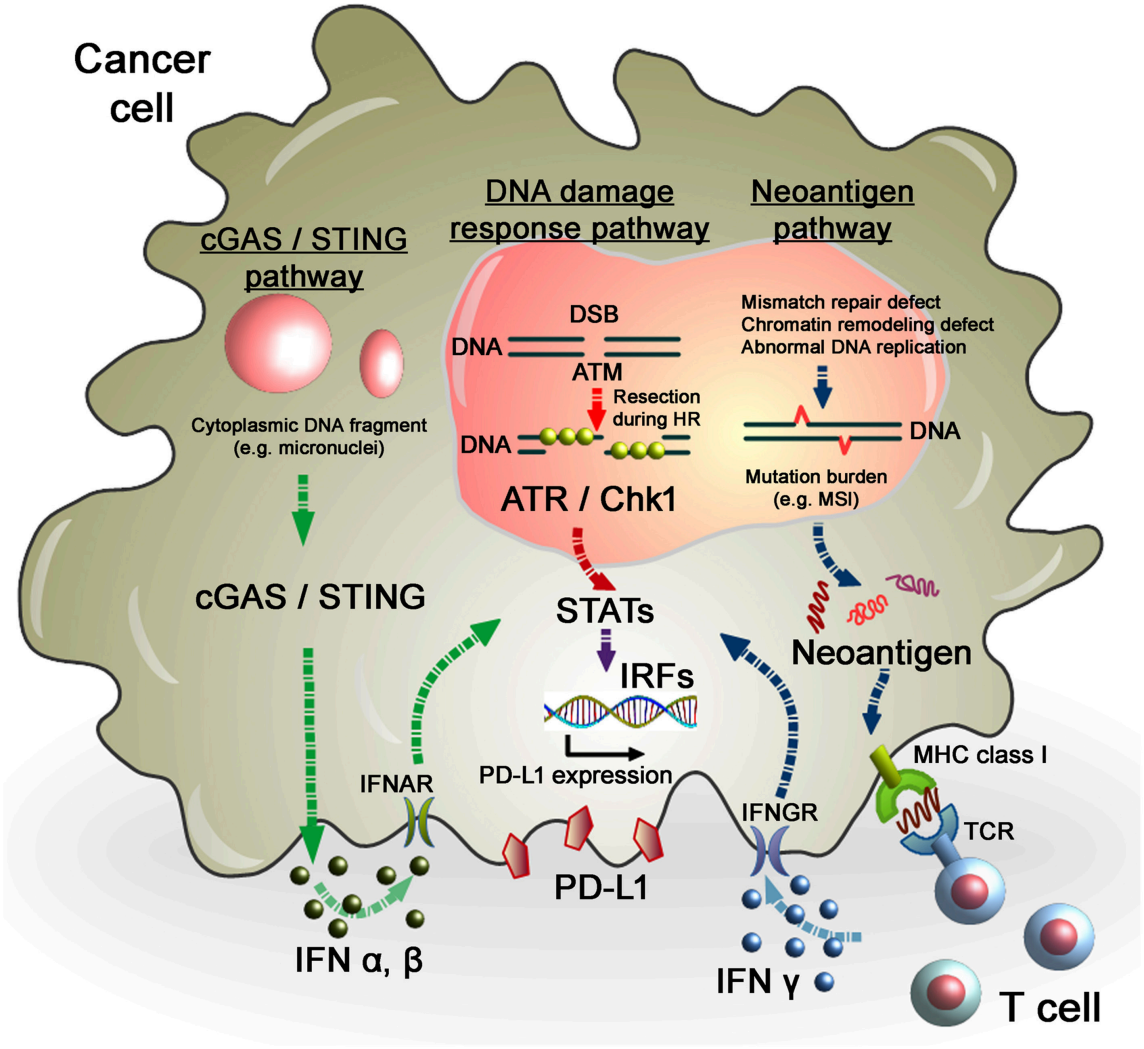
Regulation of PD-L1 expression in response to DNA damage in cancer cells. As per the DNA damage response pathway, DNA damage induced by IR or chemotherapeutic regents activates ATM/ATR/Chk1 signals, followed by the STAT-IRF pathway. In this pathway, STAT1/3-IRF1 appears to play an important role in PD-L1 upregulation after DNA damage. Alternatively, PD-L1 expression is regulated by the neoantigen pathway in the context of DNA damage and repair in cancer cells. Levels of mutation burden are associated with MSI. Mutation burdens and MSI are augmented by the defect of mismatch repair, chromatin remodeling, or abnormal DNA replication. Neoantigens presented by MHC class I, which is recognized by T cell receptors, activate T cells, followed by the release of IFNγ. IFNγ stimulates the STAT-IRF pathway via the IFNγ receptor (IFNGR) and upregulates PD-L1 expression in cancer cells. Another novel pathway, the cGAS/STING pathway, may also be involved in the activation of the IFN-STAT/IRF-PD-L1 pathway. Cytosolic DNA fragments induced by DNA damage activate the cGAS/STING pathway. Activation of the cGAS/STING pathway induces IFN type I (IFNα and IFNβ), which is incorporated into cancer cells via the IFNα/β receptor (IFNAR). IFNα/β-dependent signaling also activates the STAT-IRF pathway, resulting in PD-L1 upregulation.

In terms of regulation of PD-L1 expression in tumors, evidence suggests that cancer cells with multiple gene mutations, i.e., high mutational loads, show a higher PD-L1 expression. Importantly, high response rates of cancers with MSI, which is caused by frequent mis-DNA-incorporation at small repetitive sequences during DNA replication and is a hallmark of genomic instability, to anti-PD-1 therapy have been reported. MSI-positive tumors presenting neoantigens promote the release of IFNγ from tumor-infiltrating lymphocytes, which enhances PD-L1 expression in tumors and immune cells (49–51). Therefore, the level of MSI in tumors is considered as a marker for the efficacy of anti-PD-1/PD-L1 therapy (Figure 2). Consistent with this notion, PD-L1-positive cells in the tumor parenchyma of desmoplastic melanomas is highly associated with an increased CD8 density in the tumor invasive margin, that contribute substantial clinical benefits of anti-PD-1/PD-L1 therapy (50). Furthermore, this notion is strongly supported by the observation that patients with MMR-deficient cancers achieved a higher rate of progression-free survival following anti-PD-1/PD-L1 therapy (52, 53). Therefore, MMR status is a potent predictive marker for the response to anti-PD-L1 therapy. High expression of PD-L1 has also been observed in DNA polymerase epsilon-mutated cancers (54). More recently, the defect of a chromatin remodeling factor ARID1A has been correlated with high MSI and mutation load across multiple human cancer types due to the attenuation of MMR activity, which eventually phenocopies the MMR-defective tumors in terms of the increased activation of the neoantigen–IFNγ/PD-L1 pathway (55). Alternatively, homologous recombination (HR)-deficient tumors and immune cells were also shown to exhibit greater neoantigen loads and expression levels of TILs and PD-1/PD-L1 (56), although the magnitude of the mutations was not as substantial as with MMR-defective tumors. This could be explained by the mechanism in the process generating mutations, i.e., defects of DNA double-strand break (DSB) repair that preferentially cause chromosomal rearrangement, such as large deletions or translocations, but not a few base deletions or base substitutions. As established by DNA repair research, a few endogenously generated DSBs occur per day (57, 58). In contrast, MMR defects or abnormal DNA replication causes base substitutions at a frequency substantially greater than that of deletions or translocations induced by DSBs. Thus, the evidence suggests that high levels of mutations and neoantigens are augmented in tumors due to the failure of MMR and/or replication errors, which promotes IFNs release and the PD-L1-dependent immunosuppressive environment in the tumor-associated surrounding cells. Thus, the mutation/neoantigen/IFNγ pathway is well-understood as a major pathway that upregulates PD-L1 expression in tumors. However, such mechanisms may not be simply applied in tumors after cancer treatment because the immune cell-associated tumor environment might be drastically changed following RT or chemotherapy (see section Perspective of Combination Therapy Between Anti-PD-1/PD-L1 Therapy and RT). Particularly after ionizing radiation (IR), as described above, because the misrepair of DSBs induced by IR does not cause substantial number of mutations, the mutational loads induced by RT may not be a significant factor in the regulation of the mutation/neo-antigen/IFN pathway. In fact, However, X-rays at a dose of 2 Gy induces only ~60 DSBs in G1 phase cells which are most likely precisely repaired even in cancer cells. Thus, the number of mutations induced by X-rays must be significantly lower than that caused by MMR or replication defects.

### PD-L1 Expression in Response to DNA Damage Signaling

While neoantigen–IFN-dependent PD-L1 upregulation in tumors has been extensively studied, the importance of DNA damage signaling in the regulation of PD-L1 expression after IR has only recently been addressed. For example, we demonstrated that PD-L1 expression in cancer cells is upregulated in response to DSBs, representing the most critical type of genotoxic damage after IR (59). This upregulation requires ATM/ATR/Chk1 kinase activities, suggesting that DNA damage signaling is among the factors controlling PD-L1 expression in cancer cells (Figure 2). Consistent with these findings, the use of a specific ATR inhibitor in mouse tumor models significantly prevented IR-induced upregulation of PD-L1, which resulted in the attenuation of IR-induced CD8+ T cell exhaustion and a stimulation of the cytotoxic activity of CD8+ T cells (60).

More in-depth research regarding the molecular factors affecting PD-L1 expression after DSB induction showed that defects to Ku or BRCA2 also augmented PD-L1 upregulation after IR. DSBs are repaired by two major pathways: non-homologous end joining (NHEJ) and HR. NHEJ repairs DSBs throughout the cell cycle in mammalian cells, while HR repairs DSBs only in the S/G2 phase following DNA replication. Despite the pro-HR environment in the S/G2 phase, current models suggest that Ku70/80 heterodimers bind rapidly to DSBs, allowing NHEJ to make the first attempt toward repair (61, 62). However, if NHEJ does not ensue, the repair pathway can be switched toward HR, which is initiated by MRE11/CtIP endonuclease activity and stimulated by BRCA1-dependent RIF1 release (61, 63, 64). The second step of resection is expanded by the exonucleases EXO1, BLM, and DNA2. Following DNA end resection, single-stranded DNA is coated by RPA, which is replaced by Rad51 to facilitate homology searching and the subsequent steps of HR. ATM is activated at two-ended DSBs at an early stage after IR, namely, before resection. Alternatively, ATR is activated following RPA recruitment on single-stranded DNA. While ATM is activated at non-resected DSB ends, ATR is activated at resected DSB ends (65). Therefore, ATR activation occurs after the progression of resection during HR. Since ATM is required to initiate resection at DSB ends after IR, resection is ATM-dependent and ATR functions downstream of ATM (66). ATR phosphorylates and activates Chk1; therefore, Chk1 activation is ATM- and ATR-dependent. The high upregulation of PD-L1 in Ku or BRCA2 depleted cells can be explained by the mechanism of Chk1 activation related to the progression of resection. It is known that the Ku heterodimer complex rapidly binds and protects DSB ends and promotes NHEJ. Therefore, depletion of the Ku complex increases resection and ATR/Chk1 signaling. Alternatively, loss of BRCA2 function fails to recruit Rad51 during HR. However, because resection and RPA loading are normal in BRCA2-depleted cells, ATR/Chk1 activity can be continuously maintained on the resected DSB ends without Rad51 loading. Thus, we propose that the regulators of ATR/Chk1 activity, rather than DSB repair proteins, are important factors that influence PD-L1 expression after DSBs (59).

ATR/Chk1 is a multi-functional protein that regulates DNA repair, signaling, and transcriptional activation. The open question is how ATR/Chk1 activates the downstream signaling in the STAT1/3–IRF1 axis. ATR/Chk1 may directly or indirectly phosphorylate STAT1/3. STATs are localized in the cytoplasm, while Chk1 is activated through its phosphorylation by ATR at DNA damage sites in the nuclei (67). Thus, Chk1 must be transported from the nucleus to the cytoplasm following its activation. Previous studies have demonstrated that activated Chk1 is transported into the cytoplasm via phosphorylation of Ser286 and Ser301 by CDK during G2/M transition (68). However, it remains unclear whether activated Chk1 on chromatin gets also transported into the cytoplasm. If this is the case, Chk1 may be able to directly phosphorylate STAT1/3. The other open question is whether the cell cycle phase is involved in PD-L1 upregulation after DNA damage. IR-dependent DSBs activate ATM throughout all phases of the cell cycle. When cells are in the S/G2 phase, the HR pathway is activated and DSBs undergo resection. Therefore, ATR/Chk1 is activated only when DSBs are induced in the S/G2 phase. Importantly, because resection is ATM-dependent, ATR/Chk1 is activated downstream of ATM. While ATR/Chk1 can be activated in the S/G2 phase, these kinases are not effectively activated in cells arrested at G1 or G0/G1. Although it remains unclear how PD-L1 expression is regulated in G0/G1-arrested cancer cells, this question should be addressed in the future because most human cancer cells are arrested in the G1 phase. Recently, we showed that PD-L1 is not effectively upregulated in primary normal human dermal fibroblasts even after exposure to 30 Gy X-rays (69). Although the precise mechanisms underlying the non-responsiveness of PD-L1 upregulation after DNA damage are unclear, signaling from Chk1 to STAT1/3 in normal cells may not be as efficient as that in cancer cells. This can be explained by the insufficient Chk1 activation in primary fibroblasts, which mainly remain in the G1 phase. As an alternative explanation, epigenetic modifications of histones, such as methylation at the promoter regions, may suppress signaling from STAT1/3 to PD-L1. Although poor responsiveness of PD-L1 upregulation after DNA damage has been shown in primary normal human dermal fibroblasts, responses in other normal tissues should be carefully examined in the future. Since the canonical IFNγ pathway activates not only PD-L1 but also MHC class I and indoleamine 2, 3-dioxygenase (IDO) via STAT/IRF signaling, the DNA damage-dependent STAT/IRF pathway may also affect such IFNγ-related molecules. Therefore, it is also of interest to clarify the similarities or differences in the regulation of downstream targets between the canonical IFNγ pathway and the DNA damage-dependent STAT/IRF pathway.

In contrast to the involvement of T cell-dependent immune responses after DNA damage, previous studies demonstrated that NKG2D ligands in cancer cells are also upregulated after DNA damage in a Chk1-dependent manner (70, 71). Since multiple distinct types of ligands on tumor cells and receptors on immune cells are concerted in a time-dependent and/or a magnitude of DNA damage dependent manner, the ligand/receptor network in response to DNA damage should be comprehensively investigated to fully understand the molecular mechanisms underlying DNA damage-dependent alterations of the immune environment.

**Figure 3:**
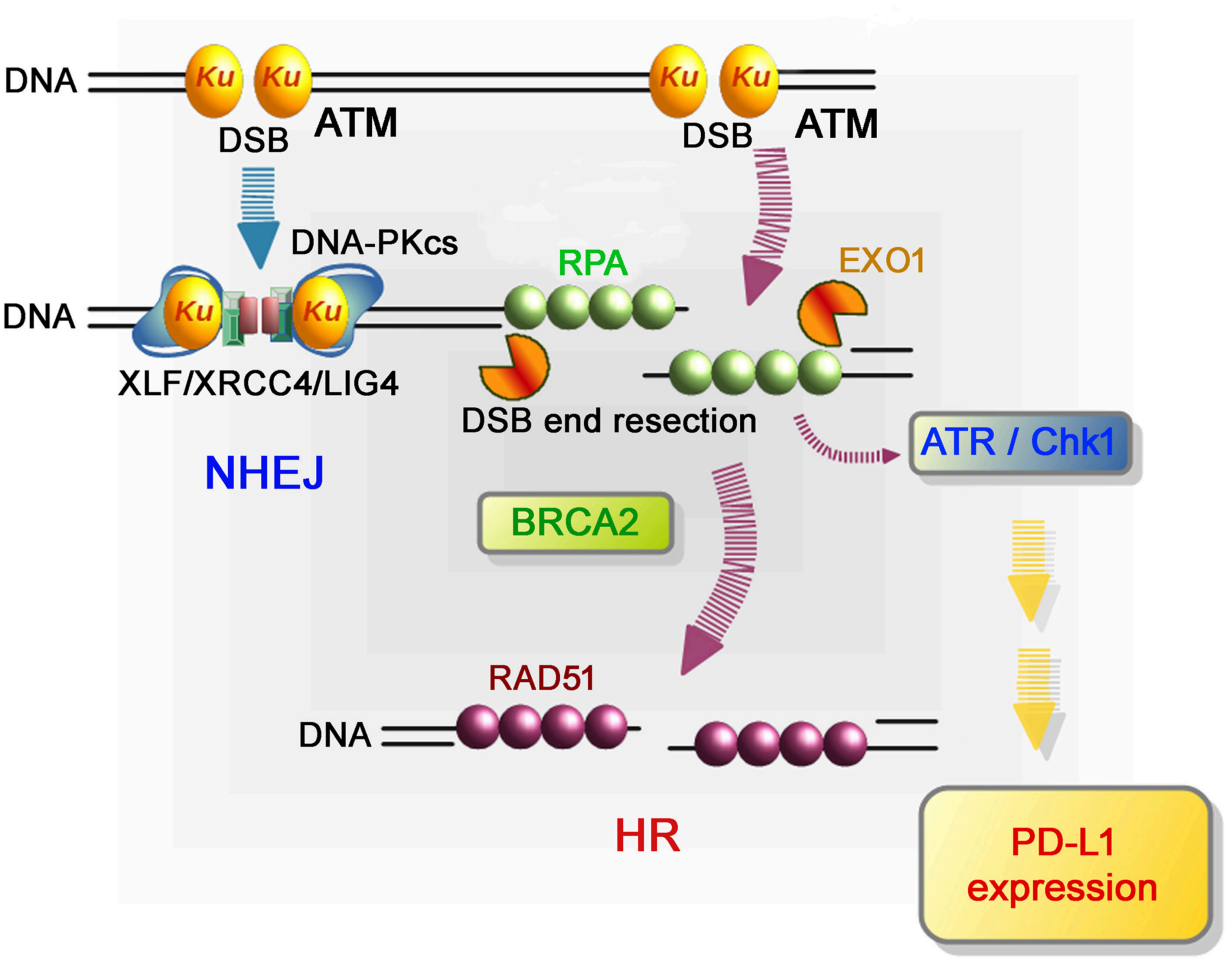
Repair pathways and signaling in response to DSB induction by IR. After DSB induction, the Ku70/80 heterodimer complex (Ku) rapidly binds to all DSB ends. Ku bound to DSB ends plays the following roles: 1) Ku70/80 complex promotes NHEJ and 2) Ku70/80 complex protects DNA ends from unscheduled digestion by DNA nucleases. In the NHEJ pathway, DSBs are rapidly rejoined by DNA-PKcs and XLF/XRCC4/LIG4 following Ku binding to DSB ends (81). On the other hand, DSB ends are digested in the 5′ to 3′ direction by EXO1 to direct repair pathway toward HR. The resected ssDNA is coated with RPA. BRCA2 promotes the protein switch from RPA to RAD51, facilitating strand invasion into the template strand for recombination-mediated repair. In terms of DNA damage signaling, ATM, which serves as a sensor of DSBs, is the major DNA damage response (DDR) kinase and is activated at unresected DSB ends. At DSB ends during HR, resection promotes a switch from ATM to ATR activation, followed by Chk1 activation. In the context of DNA damage-dependent PD-L1 expression, the activation of Chk1 is a critical step leading to STAT/IRF-mediated PD-L1 upregulation.

In contrast to the regulation of PD-L1 via ATM/ATR/Chk1 signaling, the cyclic-GMP-AMP (cGAMP) synthase (cGAS)/stimulator of interferon genes (STING)-IFNs pathway is also able to activate the immune response in tumors after DNA damage (Figure 2). Detection of cytoplasmic DNA is a fundamental mechanism of the innate immune system to sense the presence of microbial pathogens (72). Such cytoplasmic DNA has also been identified in cancer cells. Cytoplasmic DNA is generated during mitosis following endogenous DNA damage or exogenous DNA damage after RT or chemotherapeutic drugs. The cGAS recognize cytosolic DNA and catalyzes the synthesis of cGAMP which functions as a second messenger that binds and activates the adaptor protein STING. Activation of the cGAS/STING pathway induces IFN type I (IFNα and IFNβ) through IRF3/NFγB-dependent transcriptional activation (73, 74). The combination of cGAMP and anti-PD-L1 antibody exerts stronger antitumor effects than either treatment alone (75). These data strongly support the notion that the cGAS/STING pathway is important for the antitumor effect of immune checkpoint blockade. Activation of STING-dependent innate immune signaling has been observed in response to DNA damage in cancer cells (76). Following the induction of DNA damage, DNA damage signaling activates checkpoint arrest of the cell cycle (77). G2/M checkpoint arrest is critically important to prevent cells with DSB entering mitosis and causing errors of mis-segregation. Failure of G2/M checkpoint arrest leads to cell cycle progression into mitosis with DSBs and the subsequent formation of micronuclei. A recent study demonstrated that micronuclei cause activation of inflammatory signaling by recognition of the cGAS/STING pathway (78, 79). Interestingly, loss of the STING pathway prevented the regression of abscopal tumors when IR and immune checkpoint inhibitors were combined in an *in vivo* mouse model (78). These findings illustrate a novel pathway where micronuclei are recognized by cGAS/STING, as an important source of immunostimulation (79). More recently, non-canonical STING signaling in the nuclei was identified in response to DNA damage (80). Dunphy et al. demonstrated that ATM activates STING via the p53/IFI16 and TRAF6 pathways, which transduce STING to IRF3/NFκB-dependent transcriptional activation in a cGAS-independent manner. In the non-canonical STING pathway, NF-κB, rather than IRF3, is activated and the ubiquitination of STING is required for signaling, which may not be required for the canonical cGAS/STING pathway. Since cGAS/STING predominantly activates IFN type I (IFNα and IFNβ) signaling and PD-L1 transcription is regulated via IFN type II (IFNγ) signaling, PD-L1 expression may be not directly regulated in this axis. In fact, our analysis showed that depletion of cGAS or STING did not affect PD-L1 upregulation in U2OS cells after X-ray exposure (unpublished observation). However, since the immunostimulatory response is orchestrated by multiple pathways, PD-L1 expression may also be influenced via the canonical or non-canonical cGAS/STING pathway, as well as the ATM/ATR/Chk1-dependent DNA damage signaling pathway, particularly *in vivo*. An overview of PD-L1 regulation in cancer cells in response to genotoxic stress in the context of DNA damage signaling is summarized in Figure 3.

Alternatively, it has also been suggested that PD-L1 expression after IR may be regulated by toll-like receptor 4 (TLR4)/myeloid differentiation primary response gene 88 (MyD88)/TIR-domain-containing adapter-inducing interferon-β (TRIF) signaling. It is known that immunogenic cell death signaling via damage-associated molecular pattern molecules (DAMPs) activates immune response. In principle, the release of high mobility group box 1 (HMGB1), which is a ligand of TLR4, from chromatin in dying cells results in the activation of the TLR4/MyD88/TRIF pathway (82). HMGB1-dependent TLR4/MyD88/TRIF activation leads to T cell activation in response to dying tumor cells (82, 83). In contrast to the role of immune-simulative effects via the TLR4/MyD88/TRIF pathway, the involvement of the TLR4/MyD88/TRIF pathway in the regulation of PD-L1 expression has also been suggested (84). Consistent with these data, a recent study has shown that the TLR4 expression level is significantly correlated with the PD-L1 expression level and poor survival of patients with NSCLC (85). Cumulatively, the HMGB1-dependent TLR4/MyD88/TRIF pathway via immunogenic cell death may also be involved in PD-L1 upregulation in response to IR.

### Perspective of Combination Therapy Between Anti-PD-1/PD-L1 Therapy and RT

In terms of *in vivo* basic research for the development of immunoradiotherapy, the findings of several studies have suggested that the blockage of PD-1/PD-L1 interactions enhances the delay of *in vivo* tumor growth in combination with IR (21, 40). Combination of anti-PD-1 antibody and stereotactic radiation improves survival in mice with intracranial gliomas (86). Stereotactic radiation therapy increase anti-PD-1 antibody dependent antitumor responses via cross-presentation of tumor antigens (87). These studies demonstrated the ability of RT to provide an additional mechanistic rationale for combining radiation with PD-1 blockade. Park and colleagues demonstrated that stereotactic ablative RT induces abscopal tumor-specific immune responses in both irradiated and non-irradiated tumors, which is potentiated by PD-1 blockade in bilateral flank mice models of melanoma and renal cell carcinoma (88). Thus, preclinical evidence indicates that combining RT with anti-PD-1 treatment increases the anti-tumoral activity of both treatments and enhances long-term survival (89).

The results of the Pacific trial showed that progression-free survival of patients with advanced NSCLC was significantly longer with durvalumab than with a placebo after concurrent chemoradiotherapy (90). Other clinical studies demonstrated that the combination of local irradiation with anti-PD-1/PD-L1 checkpoint blockade was a feasible and synergistic treatment for cancer and improved patient outcomes (91, 92). Secondary analysis of the phase 1 KEYNOTE-001 trial suggested that previous treatment with RT in patients with advanced NSCLC resulted in longer progression-free survival and overall survival with pembrolizumab treatment than that of patients who did not receive previous RT (93). It is a fact that 25% of patients with melanoma showed regression of non-irradiated lesions when anti-PD1 was continued after RT to a tumor site that had progressed upon anti-PD1 monotherapy (94).

To date, although ~100 clinical trials of anti-PD-1/PD-L1-based immunoradiotherapy are ongoing (~20 active trials are listed in Table 2), little is known about whether fractionation regimens, radiation dosages, and the timing of IR have any specific impact on the antitumor immune response (95). While data suggest that single fraction radiation is better than multiple fractions for inducing antitumor immunity (96), other studies report that both single and multiple fractions had similar effects on antitumor immunity (97). In addition to this, fractionated, but not single-dose RT induced an immune-mediated abscopal effect to maximize tumor immunity (98, 99). Regarding an approximate dose in immunoradiotherapy, Vanpouille-Box, and colleagues reported that the DNA exonuclease trex1 is induced by a radiation dose >12–18 Gy in different cancer cells and attenuates cGAS/STING mediated immunogenicity by degrading DNA that accumulates in the cytosol upon radiation (100). Regarding the timing of combination, Dovedi et al. reported that concurrent checkpoint blockade immunotherapy starting on the day of or during fractionated RT was better than starting checkpoint blockade immunotherapy after the completion of RT (21).

**Table 2:** Active clinical trials of anti-PD-1/PD-L1 antibody treatment combining with radiotherapy or chemoradiotherapy.

**COMBINED WITH RADIOTHERAPY**						
**Conditions**	**Study title**	**Drug**	**RT**	**Concurrent chemo**	**Phase**	**NCT number**
Melanoma	A Pilot Study to Evaluate the Safety and Efficacy of Combination Checkpoint Blockade Plus External Beam Radiotherapy in Subjects With Stage IV Melanoma	Nivolumab and ipilimumab	EBRT 30 Gy in 10 fractions 27 Gy in 9 fractions	N/A	1	NCT02659540
	A Phase II Trial of Stereotactic Body Radiotherapy With Concurrent Anti-PD1 Treatment in Metastatic Melanoma.	Anti-PD-1 treatment	SBRT 24 Gy in 3 fractions	N/A	2	NCT02821182
	Trial of Pembrolizumab and Radiotherapy in Melanoma	Pembrolizumab	24 Gy in 3 fractions	N/A	2	NCT02562625
Non-small Cell Lung Cancer	A Pilot Study of MPDL3280A and HIGRT (Hypofractionated Image-Guided Radiotherapy) in Metastatic NSCLC	Atezolizumab (MPDL3280A)	Hypofractionated Radiotherapy	N/A	1	NCT02463994
	Pembrolizumab After SBRT vs. Pembrolizumab Alone in Advanced NSCLC	Pembrolizumab	SBRT 24 Gy in 3 fractions	N/A	2	NCT02492568
Head and Neck Squamous Cell Carcinoma	Pembrolizumab + Radiation for Locally Adv SCC of the Head and Neck (SCCHN) Not Eligible Cisplatin	Pembrolizumab	IMRT	N/A	2	NCT02609503
	Tolerance and Efficacy of Pembrolizumab or Cetuximab Combined With RT in Patients With Locally Advanced HNSCC	Pembrolizumab	EBRT	N/A	2	NCT02707588
Glioblastoma	Phase 2 Study of MEDI4736 in Patients With Glioblastoma	Durvalumab (MEDI4736)	EBRT	N/A	2	NCT02336165
Diffuse Intrinsic Pontine Glioma	A Phase I/ II Clinical Trial of MDV9300 (Pidilizumab) in Diffuse Intrinsic Pontine Glioma	Pidilizumab (MDV9300)	EBRT	N/A	1/2	NCT01952769
Metastatic Colorectal Cancer	Assess the Efficacy of Pembrolizumab Plus Radiotherapy or Ablation in Metastatic Colorectal Cancer Patients	Pembrolizumab	EBRT/Radiofrequency ablation	N/A	2	NCT02437071
Metastatic breast cancer	Study to Assess the Efficacy of Pembrolizumab Plus Radiotherapy in Metastatic Triple Negative Breast Cancer Patients	Pembrolizumab	EBRT	N/A	2	NCT02730130
Metastatic Urothelial Cancer	Trial of Stereotactic Body Radiotherapy With Concurrent Pembrolizumab in Metastatic Urothelial Cancer	Pembrolizumab	SBRT	N/A	1	NCT02826564
Head and Neck Cancer Renal Cell Cancer Melanoma Lung Cancer	Radiation Therapy and MK-3475 for Patients With Recurrent/Metastatic Head and Neck Cancer, Renal Cell Cancer, Melanoma, and Lung Cancer	Pembrolizumab (MK-3475)	EBRT 8 Gy in 1 fraction 20 Gy in 4 fractions	N/A	1	NCT02318771
**COMBINED WITH CHEMORADIOTHERAPY**						
Head and Neck Squamous Cell Carcinoma	Safety Testing of Adding Nivolumab to Chemotherapy in Patients With Intermediate and High-Risk Local-Regionally Advanced Head and Neck Cancer	Nivolumab	IMRT 70 Gy in 35 fractions	Cisplatin or Cetuximab	1	NCT02764593
Non-Small Cell Lung Cancer	MPDL3280A With Chemoradiation for Lung Cancer	MPDL3280A (Atezolizumab)	EBRT 60–66 Gy in 30–33 fractions	carboplatin and paclitaxel	2	NCT02525757
	Nivolumab Combination With Standard First-line Chemotherapy and Radiotherapy in Locally Advanced Stage IIIA/B Non-Small Cell Lung Carcinoma	Nivolumab	EBRT	platinum-based	2	NCT02434081
	Cisplatin and Etoposide Plus Radiation Followed By Nivolumab/Placebo For Locally Advanced NSCLC	Nivolumab	3DCRT/IMRT	Cisplatin and Etoposide	3	NCT02768558
Malignant Glioma	Hypofractionated Stereotactic Irradiation (HFSRT) With Pembrolizumab and Bevacizumab for Recurrent High Grade Gliomas	Pembrolizumab	Hypofractionated Stereotactic Irradiation	Bevacizumab	1	NCT02313272
Glioblastoma	Radiation Therapy With Temozolomide and Pembrolizumab in Treating Patients With Newly Diagnosed Glioblastoma	Pembrolizumab	EBRT	Temozolomide	1/2	NCT02530502
Advanced Cancer	Study of REGN2810 (Anti-PD-1) in Patients With Advanced Malignancies	Cemiplimab (REGN2810)	Hypofractionated radiotherapy	Cyclophosphamide	1	NCT02383212

As described above, immunoradiotherapy is highly promising, particularly for non-responders to anti-PD-1/PD-L1 monotherapy. Accumulating evidence suggests that RT activates host immunity. However, because PD-L1 is upregulated, as well as other immunosuppressive response [see section Immunosuppressive Effects of Radiotherapy (RT)], in response to DNA damage signaling after IR, the immune response may not be fully activated after RT. Therefore, anti-PD-1/PD-L1 treatment is likely able to normalize the appropriate activation of the immune response under the immunosuppressive conditions after IR. Immune activation induced by RT can be categorized as follows. IR triggers immunogenic cell death, which is characterized by the release of danger signals to ensure the effective presentation of tumor antigens and priming of antigen-specific T cells (101). Damage-associated molecular patterns, such as ATP, calreticulin, heat shock proteins (HSPs), and HMGB1, are released or expressed upon the immunogenic cell death. For example, ATP attracts DCs into the tumor (102) and the cell surface exposure of calreticulin, which is an endoplasmic reticulum-resident protein, promotes phagocytosis of irradiated tumor cells (103, 104). In addition, IR induces cancer cell-death that releases HMGB1 (105). Furthermore, HMGB1 activates DCs via binding to TLR4 (106, 107). Both calreticulin and HMGB1 were found to be essential for the antigen-specific T-cell responses in murine tumor models (108). IR also enhances the immune response via cross-presentation of tumor antigens. Gupta et al. reported that local high-dose RT resulted in the activation of tumor-associated DCs in B16gp tumor-bearing C57BL/6 mice (109). Enhancement of antigen-presenting cell function and tumor immunity through signaling pathways, such as NFκB, has been demonstrated after DNA damage (110). As described in the previous section, the type I IFN pathway is upregulated via the cGAS/STING pathway after IR (100). The release of type I IFN activates DCs, which is followed by immunoactivation (111, 112).

The immunosuppressive environment in tumors is established through the obstruction of immune cell infiltration and/or growth of tumor-infiltrating lymphocytes as well as some other factors as described in section Immunosuppressive Effects of Radiotherapy (RT) (113). Therefore, re-acquisition of T cell infiltration in tumor tissue promotes the restoration of an adequate immune response against tumors. Among the multiple approaches suggested to restore an adequate immune response, IR is believed to overcome the immunosuppressive environment by promoting T-cell infiltration to tumors (114). Lower doses of radiation therapy normalize dysfunctional tumor vasculature, thereby allowing the infiltration of antigen-specific T cells into the tumor tissue and the mediation of antitumor effects (114). Hallahan and colleagues showed that radiation increased the expression of E-selectin and ICAM-1 in human endothelial cells (115). The release of the chemokine CXCL16, which can attract CXCR6-expressing CD8 T cells to tumor tissues, and CXCL21 by irradiation was reported (116, 117). The axis of MHC class I pathway has been identified as another route of immune activation after IR. Many cancers have significant correlations between poor clinical prognosis and the low expression of MHC class I molecules. Thus, the downregulation of MHC class I molecules may impede the detection of cancer cells under immunosuppressive conditions (118, 119). Accumulating studies demonstrated the changes in MHC class I expression and antigen presentation that occur after IR. Reits et al. reported that irradiation led to a dose-dependent increase in the levels of intracellular peptides and increased protein synthesis via mTOR activation, resulting in an increase in MHC class I expression (118). Also, we previously reported that HLA class I upregulation by preoperative hyperthermo-chemoradiotherapy in patients with rectal cancer (119). In addition to the T-cell dependent pathway, IR also upregulates the NK pathway via activation of NKG2D ligands, which are potent immunomodulators of the innate and adaptive immune responses (70, 71, 120–122). The increase in antigen presentation and expression of MHC class I molecules, together with the immunogenic release of damage-associated molecular patterns, is believed to highly contribute to the enhanced susceptibility of irradiated cells, which results in immune-mediated cancer cell death (118). Taken together, the evidence strongly suggests that anti-PD-1/PD-L1 treatment is effectively able to normalize the appropriate immune response against tumors in combination with the immune activation by IR.

In addition to the development of immunotherapy, RT has been largely advanced by introducing novel technologies, such as heavy ion particle and proton therapy. As the next generation of immunoradiotherapy, the results of clinical trials of anti-PD1/PD-L1 and heavy ion particle therapy have been highly promising. Although little is known about the immune response following heavy ion particle irradiation, a combination may be the best choice because heavy ion particle therapy has two cooperating advantages as compared with other radiotherapies, i.e., high-specificity against tumors and greater cell killing effects. Particularly, because the great cell killing effect is related to the type of DNA damage, which can be defined as complex DNA lesions and clustered DSBs (Hagiwara et al., JRR, in press), heavy ion-specific DNA damage may cause greater immune activation, although PD-L1 may be also highly upregulated. However, importantly, even if PD-L1 is highly upregulated after heavy ion particle therapy, the downregulation of immune activity should be normalized by treatment with anti-PD1/PD-L1 antibodies.

## Combination of the Radiation and Other Immunotherapeutic Treatment Modalities

One of the potential systemic treatment modalities is the addition of immunotherapeutic methods to standard RT including vaccines, cytokines, and cytokine inducers, adoptive cell transfer (DCs, NK cells, T cells). Thus, radiation-induced biological effects in the tumor could make tumors more susceptible to immune-mediated responses. Application of the vaccine with various immunostimulatory adjuvants could promote the activation of T cells toward antigens released by RT. Thus, Chakraborty et al. in the preclinical study demonstrated that combination of the local radiation (single 8 Gy irradiation) with a recombinant vaccine and fowlpox viruses containing the human CEA gene, the murine ICAM-1, leukocyte function associated antigen 3 genes, and B7-1 could elicit a tumor-specific CD4+ and CD8+ T cell responses in the mouse model of MC38 colon adenocarcinoma (123). Subsequent randomized phase II clinical trial utilized RT with poxviral vaccine encoding prostate-specific antigen (PSA) and the co-stimulatory molecule B7.1 showed a development of T-cell specific response to multiple tumor-associated antigens (including MUC-1, PSMA, PSCA, PAP) (124). Intriguingly, in the presented study the designed vaccine against a single antigen could induce immune responses with formation *de novo* of T cell specific for antigens not presented in the vaccine. This indicates that a vaccine treatment could augment the RT-induced tumor killing that could have a potential role in metastatic tumors eradication due to the induction of polyclonal immune response.

Alternative for vaccination to promote cross-priming of tumor infiltrating lymphocytes (TILs) could be an application for denritic cells (DCs). Few clinical trials reported of the successful combination of intratumoral delivery of DCs and RT (125–127). Thus, combination of 45 Gy RT with intra-prostatic administration of DCs resulted in prostate CD8+ T cell increase in the TILs of the localized prostate cancer in HLA-A2(+) patients (126). Similar results were reported for the cohort of soft tissue sarcoma patients when 9 patients (52.9%) developed tumor-specific immune responses and 12 of 17 patients (70.6%) were progression free after 1 year (127). To further boost DC activation other immunostimulators could be applied including:

- ***TLR2 agonists*** [e.g., protein-bound polysaccharide (PSK) from *Basidoiomycete coriolus versicolor*, IMM-101 (heat killed *Mycobacterium obuense*), arabinomannan extracted from *Mycobacterium tuberculosis* strain Aoyama B (Z-100)] (128–133)
- ***TLR3 agonists*** [e.g., poly-ICLC (synthetic double stranded RNA)] (134, 135)
- ***TLR7 agonists*** (e.g., imiquimoid) (136)
- ***TLR9 agonists*** (e.g., CpG DNA PF-3512676) (137)
- ***Cytokines*** (e.g., GM-CSF, fms-like tyrosine kinase-3 (flt-3) ligand) (138).

Although these stimulators demonstrated promising therapeutic potential in preclinical and early phase clinical trials, a phase III trial of the immunomodulator Z-100 for stage IIB-IVA cervical cancer (JGOG study) showed a trend for improvement on OS, although the statistical power was less than anticipated because survival rates were unexpectedly higher for both arms (133).

Among other immunostimulators, the application of cytokines might improve the effector function of the TILs. Several clinical trials demonstrated the potency of the various cytokines (e.g., IFN-α, TNF-α, IL-2) combined with RT in pancreatic cancer, melanoma, renal cell carcinoma (139–144). However, employed cytokines induced severe toxicities in the patients that significantly limited the application of these adjuvants in clinical trials.

## Conclusions

Reported clinical trials combining immunotherapy and RT demonstrated a therapeutic potency in treatment of various cancer types due to the augmentation of the immune-mediated responses. However, the observed effects were modest because of immunosuppressive effects. Cells of the tumor micromilieu (e.g., TAMs, MDSCs, Tregs, etc) significantly hamper T cell activity in the tumor. Presumably editing tumor microenvironment could further improve the existing treatment schemes. One approach could be based on application of modulators of certain suppressive cells. Thus, cyclophosphamide when applied in low doses can selectively deplete Tregs and thereby improve anti-tumor immune responses (145). More recently tadalafil, the phosphodiesterase (PDE)-5 inhibitor, was shown to impair MDSCs functions and enhance antitumor immunity in advanced melanoma (146) and head and neck squamous cell carcinoma patients (HNSCC) (147). Indeed stable disease melanoma patients displayed higher numbers of CD8+ T cells in the center of metastasis compared to patients with progressed disease (146).

Immune checkpoint inhibitors, particularly inhibitors of PD-L1/PD-1 axis, can also enhance effector cell function and data from preclinical and clinical trials showed significant improvement in overall survival of patients of several types of cancers. Recent studies demonstrate that two pathways, i.e., mutational loads-IFNγ pathway and DNA damage signaling pathway, are involved in the regulation of PD-L1 expression in tumors. Although the signaling of PD-L1 expression in tumors is comprehensively regulated, particularly in combination with RT, the understanding of the mechanism underlying PD-L1 expression in response to DNA damage would be important to provide the basis for the combined therapies and promote personalized immuno-radiotherapy. In conclusion, immuno-radiotherapy is highly promising, particularly for non-responders to inhibitors of PD-L1/PD-1 pathway. Introduction of new radiotherapeutic technologies, such as heavy ion particle or proton therapy, might further improve the effects of immunotherapy.

## Data Availability

The datasets generated for this study are available on request to the corresponding author.

## Author Contributions

The manuscript was conceived and designed by MS, HS, GM, and AS. All authors wrote and revised the manuscript.

### Conflict of Interest Statement

The authors declare that the research was conducted in the absence of any commercial or financial relationships that could be construed as a potential conflict of interest.
